# Salvianolic acid B protects against lipopolysaccharide-induced behavioral deficits and neuroinflammatory response: involvement of autophagy and NLRP3 inflammasome

**DOI:** 10.1186/s12974-017-1013-4

**Published:** 2017-12-06

**Authors:** Pei Jiang, Yujin Guo, Ruili Dang, Mengqi Yang, Dehua Liao, Huande Li, Zhen Sun, Qingyan Feng, Pengfei Xu

**Affiliations:** 1 Institute of Clinical Pharmacy & Pharmacology, Jining First People’s Hospital, Jining Medical University, Jining, China; 2 Department of Pharmacy, Hunan Cancer Hospital, Central South University, Changsha, China; 3 Institute of Clinical Pharmacy & Pharmacology, Second Xiangya Hospital, Central South University, Changsha, China; 4 Department of Neurology, Jining First People’s Hospital, Jining Medical University, Jining, China

**Keywords:** Autophagy, Neuroinflammation, NLRP3, Salvianolic acid B, Depression

## Abstract

**Background:**

The NLRP3 inflammasome activation and neuroinflammation are known to be involved in the pathology of depression, whereas autophagy has multiple effects on immunity, which is partly mediated by the regulation of inflammasome and clearance of proinflammatory cytokines. Given the emerging evidence that autophagy dysfunction plays an essential role in depression, it is very likely that autophagy may interact with the inflammatory process in the development and treatment of depression. Salvianolic acid B (SalB), a naturally occurring compound extracted from *Salvia miltiorrhiza*, contains anti-inflammatory and antidepression properties and has recently been proven to modulate autophagy. In this study, we sought to investigate whether autophagy is involved in the inflammation-induced depression and the antidepressant effects of SalB.

**Methods:**

The effects of prolonged lipopolysaccharide (LPS) treatment and SalB administration on behavioral changes, neuroinflammation, autophagic markers and NLRP3 activation in rat hippocampus were determined by using behavioral tests, real-time PCR analysis, western blot, and immunostaining.

**Results:**

Our data showed that periphery immune challenge by LPS for 2 weeks successfully induced the rats to a depression-like state, accompanied with enhanced expression of pro-inflammatory cytokines and NLRP3 inflammasome activation. Interestingly, autophagic markers, including Beclin-1, and the ratio of LC3II to LC3I were suppressed following prolonged LPS exposure. Meanwhile, co-treatment with SalB showed robust antidepressant effects and ameliorated the LPS-induced neuroinflammation. Additionally, SalB restored the compromised autophagy and overactivated NLRP3 inflammasome in LPS-treated rats.

**Conclusions:**

Collectively, these data suggest that autophagy may interact with NLRP3 activation to contribute to the development of depression, whereas SalB can promote autophagy and induce the clearance of NLRP3, thereby resulting in neuroprotective and antidepressant actions.

## Background

Depression is the most common debilitating psychiatric disease with lifetime prevalence of more than 10%. Overall, currently available antidepressants are effective, but almost 50% of patients fail to reach sustained remission and 20% of these patients do not respond to any intervention [1]. The poor effectiveness of antidepressant treatment is at least partially attributed to the fact that the molecular mechanisms involved in the aetiology of the disorder and in the therapeutic actions of the antidepressants are still largely unknown.

Autophagy is an evolutionarily homeostatic cellular process, which can sense intracellular stress and rapidly mount a response to cope with the damage through sequestration and degradation of damaged organelles and compromised proteins. This process is orchestrated by a series of autophagy-related genes (Atg genes), such as Beclin-1 and LC3-II (derived from LC3-I upon lipidation), which are reliable biomarkers for assessing autophagy process [2]. As a housekeeping pathway and guardian of cellular homeostasis, autophagy is involved in a myriad of brain functions. The disturbance in autophagy is associated with several neurodegenerative and mood disorders [3, 4]. Recently, multiple lines of evidence reveal a potential role of autophagy in depression. Beyond the impact on monoaminergic neurotransmission, many antidepressants, such as selective serotonin reuptake inhibitors (SSRIs), can facilitate autophagy flux and induce the expression of autophagy markers [5, 6]. Moreover, the mTOR inhibitor, rapamycin, confers antidepressant effects through promoting autophagy, indicating that autophagy is essentially involved in the antidepressant action [7]. However, the potential mechanisms by which the autophagy process may contribute to both the development and treatment of depression remain equivocal.

Evolving evidence consistently reveals a close linkage between inflammation and major depressive disorder [8]. Immune system activation, such as exposure to the bacterial endotoxin lipopolysaccharide (LPS), also induces sickness behavior in animals, which resembles depressive-like symptoms, including fatigue and anhedonia, whereas autophagy actively participate in the clearance of inflammasomes and proinflammatory cytokines, thus functioning as a central fulcrum that balances inflammatory responses [9]. The Nod-like receptor pyrin containing 3 inflammasome (NLRP3) is an intracellular multiprotein complex detecting a series of substances emerging during infections, cellular damage, or metabolic disturbances and is recently indicated as a central mediator of both stress and LPS-induced depression [10]. By sensing a bunch of divergent invading pathogens and cellular damage self-danger signals, NLRP3 interacts with the apoptosis-associated speck-like protein containing a caspase recruitment domain (ASC) which recruits pro-caspase-1 to form a large protein complex, called the NLRP3 inflammasome. This process results in autocleavage of caspase-1, which proteolytically processes pro-IL-1β into its mature form. The cleaved IL-1β may further activate nuclear factor kappa B (NF-κB) pathway to mediate transcription and function of other inflammatory cytokines, thereby inducing brain innate immunity and inflammation [11]. While depression is tightly related to the hyperactivation of NLRP3 inflammasome and overproduction of IL-1β, autophagy removes aggregated inflammasome structures, thus contributing to dampening proinflammatory responses [12]. Based on these clues, it is reasonable to hypothesize that the interrelationship between autophagy and neuroinflammation process, especially NLRP3 activation, may play a pivotal role in the progression of depression.

Salvianolic acid B (SalB), a naturally occurring compound extracted from *Salvia miltiorrhiza*, contains anti-inflammatory and antidepression properties and has recently been proven to modulate autophagy [13, 14]. Therefore, in the present study, we sought to investigate the neuroprotective and neuroimmune modulating effects of SalB in LPS-induced depression animal model, and whether autophagy is involved in the inflammation-induced depression and the antidepressant effects of SalB.

## Methods

### Animals

Male, Sprague-Dawley rats (200–230 g) were housed under standard conditions of temperature (23 ± 2°C) and light (12:12-h light/dark cycle), with free access to food and water.

### Drug treatment

Rats were randomly allocated to one of the four groups (*n* = 8): control, SalB, LPS and LPS + SalB. While the rats in SalB group were intraperitoneally injected with 20 mg/kg SalB (Lvgu Biotechnology, China), the control group received same volume of saline. The rats received LPS (*Escherichia coli* serotype0111:B4, Sigma-Aldrich) via intraperitoneal injection at a dose of 500 μg/kg every 2 days for a total of seven injections. The dose and treatment duration of LPS were chosen to effectively provoke depressive-like behavior without causing immuno-tolerance based on our previous research [15].The animals in LPS + SalB group received daily SalB treatment in addition to the LPS treatment regimen. Body weight of these rats was monitored throughout the experiment, and the drug doses were adjusted accordingly. To further evaluate the impact of prolonged LPS exposure on autophagic flux in vivo, another experiment was conducted and the animals were divided into four groups (*n* = 6): control, control + chloroquine (CQ), LPS, and LPS + CQ. The treatment protocol of LPS was the same as the above-mentioned and the lysosomal inhibitor, CQ (50 mg/kg) (Sigma, USA, dissolved in a 5% DMSO solution), was intraperitoneally pre-administered to the animals before the last injection of LPS. At the same time, an equal volume of DMSO was injected for the controls.

### Forced swim test (FST)

The paradigm is based upon the evaluation of immobility, as a measure of behavioral despair in stressful and inescapable situations [16]. In brief, each rat was placed in a Plexiglas cylinder (45-cm height, 25-cm diameter) containing approximately 35 cm of water (24 ± 1 °C) for 15 min. The rats were then dried and removed to their home cage. They were placed again in the cylinders 24 h later, and a 5-min swim test was conducted. Each test session was videotaped, and the duration of immobility, which is defined as floating passively and only making slight movements to keep the head above water, was scored by an experienced observer blind to the experiment design.

### Sucrose preference test (SPT)

SPT is widely used for the measurement of stress-induced anhedonia state, a key depressive-like behavior in rats [17]. Prior to SPT, all the rats were housed individually and habituated to 48 h of forced 1% sucrose solution consumption in two bottles on each side. Then, after 14-h water deprivation, we placed two pre-weighed bottles, one containing 1% sucrose solution and another containing tap water to each rat. The side (left and right) of the two bottles was randomly placed in order to avoid spatial bias. The bottles were weighed again after 1 h, and the weight difference was considered to be the rat intake from each bottle. The preference for sucrose was measured as a percentage of the consumed 1% sucrose solution relative to the total amount of liquid intake.

### Elevated plus maze (EPM) test

EPM test was performed to evaluate the LPS-induced anxiety-like behavior in rats [18]. In brief, the maze apparatus was a cross-shaped Plexiglas platform with two opposite open arms (OA, 50 × 10 cm) and two opposite closed arms (CA, 50 × 10 cm) with 40-cm walls, connected by a central platform (CP, 10 × 10 cm) and elevated 50 cm from the floor in a dimly lit room. The animals were placed at the center of the apparatus with its head facing towards an open arm. The total number of entries into the open and closed arms, and the time spent in each arm during the 5-min period were recorded with a video camera mounted vertically above the apparatus.

### Western blot analysis

One day after the behavioral tests, the rats were anesthetized with 10% chloral hydrate (4 mL/kg) and the tissues were rapidly collected. For western blotting analysis, total protein was prepared from the right hippocampus, and the protein concentrations were analyzed using Bradford method. The samples were loaded on precast 12% SDS-PAGE gels with approximately 50 μg protein in each lane. The following antibodies and concentrations were used over night at 4 °C; LC3 (Cell signaling, 4108; 1:1000), Beclin-1 (Abcam, ab62557; 1:1000), NLRP3 (Abcam, ab214185; 1:200), ASC (Abcam, ab175449; 1:1000), caspase-1 (Abcam, ab1872; 1:1000), IL-1β (Abcam, ab9722; 1:1000), and β-actin (Proteintech, 66009-1-Ig; 1:4000). The signals were normalized to β-actin as an internal standard. It was then probed with HRP-conjugated secondary antibody for 40 min. The film signals were digitally scanned and then quantified using ImageJ software. The signals were normalized to β-actin as an internal standard.

### Real-time PCR analysis

Total RNA was extracted by using Trizol reagent (Invitrogen, USA) from the right hippocampal homogenates following the manufacturer’s instructions. Quantitative PCR was performed on Bio-rad Cx96 Detection System (Bio-rad, USA) using SYBR green PCR kit (Applied Biosystems, USA) and gene-specific primers (Table 1). Each cDNA was tested in triplicate. Thermoprofile conditions were 50 °C for 2 min, 95 °C for 10 min, and 40 cycles of amplification at 95 °C for 15 s and 60 °C for 1 min. Relative quantitation for PCR product was normalized to β-actin as an internal standard.

**Table 1:** Primer sequences used for the qPCR analysis

Gene	Sense primer (5′-3′)	Antisense primer (5′-3′)	Amplicon length (bp)
IL-1β	AGGTCGTCATCATCCCACGAG	GCTGTGGCAGCTACCTATGTCTTG	119
IL-6	CACAAGTCCGGAGAGGAGAC	ACAGTGCATCATCGCTGTTC	167
TNF-α	GAGAGATTGGCTGCTGGAAC	GAGAGATTGGCTGCTGGAAC	82
β-Actin	CATCCTGCGTCTGGACCTGG	TAATGTCACGCACGATTTCC	116

### Histopathological staining

Brains were collected, and the left hippocampus was rapidly dissected from the representative animals in each group. The hippocampus was fixed in 10% phosphate-buffered paraformaldehyde and then embedded in paraffin, prepared for histopathological examination and immunohistochemical staining. The paraffin tissue blocks were prepared for sectioning at 5-μm thickness by sledge microtome. The obtained tissue sections were stained by hematoxylin and eosin (H&E).

### Immunostaining

For the immunohistochemical analysis, paraffin-embedded coronal sections of the hippocampus (6-μm thickness) were dewaxed in xylol, rehydrated, and rinsed in phosphate-buffered saline (PBS). Subsequently, the sections were blocked with 3% H_2_O_2_ for 20 min. Antigen retrieval was performed by boiling the sections on an electric stove in a citric acid buffer (0.01 mol/L, pH 6.0), followed by incubation with blocking 5% goat serum for 1 h at room temperature. After incubated with the NLRP3 primary antibody (Abcam, ab214185; 1:200) overnight at 4°C, the sections were incubated with 2-step plus®Poly-HRP AntiMouse/Rabbit IgG Detection System (ZSGB-BIO, China) against the primary antibodies. Finally, the sections were developed with 3,3′-diaminobenzidine (DAB) and were counterstained with hematoxylin. For immunofluorescent staining, without blocking endogenous peroxidase, the sections were incubated with the primary antibodies anti-IBA-1 (Abcam, ab15690; 1:200) or anti-LC3 (Cell signaling, 4108; 1:100). After washes, Cy3-Labeled goat anti-Mouse IgG antibodies (Beyotime Biotechnology, China, A0521; 1:1000) or Cy3-Labeled goat anti-rabbit IgG antibodies (Beyotime Biotechnology, China, A0516; 1:1000) were applied for 1 h. The sections were washed with PBS three times and stained with DAPI (Beyotime Biotechnology, China, C1006) to stain the cell nuclei. Immunofluorescent images were taken with an inverted fluorescence microscope (IX53, Olympus, Japan).

### Statistical analysis

Results from the experiment were expressed as means ± SD and analyzed using SPSS version 13.0 software. Normality of distribution was assessed by the Lilliefors test, and homogeneity of variance was tested with the Levene’s test. Differences between groups were determined by one-way analysis of variance (ANOVA) test, followed by Tukey’s test for post hoc comparisons when equal variances were assumed. The prior level of significance was established at *p* < 0.05.

## Results

### Behavioral tests

**Fig. 1:**
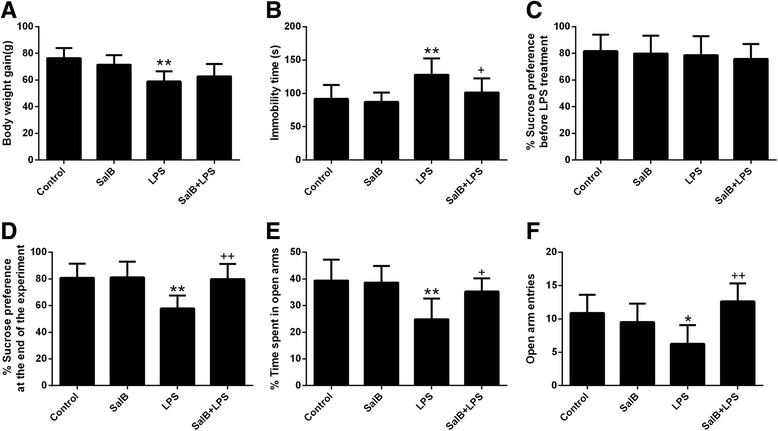
SalB alleviates LPS-induced behavioral changes. Effect on SalB and LPS on body weight changes (**a**). Depression-like behaviors was assessed by forced swimming test (**b**) and sucrose preference test (**c**, **d**). Anxiety-like behaviors was assessed by the elevated plus maze test (**e**, **f**). Data are means ± SD (*n* = 8). ^*^ *p* < 0.05, ^**^ *p* < 0.01 compared to the control group. ^+^ *p* < 0.05, ^++^ *p* < 0.01 compared to the LPS group

Animals exposed to LPS showed significantly less weight gain compared with control group (*p* < 0.01), and SalB treatment had no effect on body weight changes (*p* > 0.05) (Fig. 1a). The LPS-treated rats also exhibited increased immobility time in FST (*p* < 0.01) (Fig. 1b) and decreased sucrose preference in SPT (*p* < 0.01) (Fig. 1c, d) compared with the control group, whereas the drug use mitigated the depressive-like behaviors in LPS-exposed rats. In line with previous findings [14], LPS challenge induced an anxiogenic effect as evident by the significant reduction in the open-arm time (Fig. 1e, *p* < 0.01) and number of entries (Fig. 1f, *p* < 0.05) in the open arm, whereas the anxiolytic effect of SalB was also observed. For the control rats, SalB alone had no effect on the behavioral profile (*p* > 0.05).

### SalB mitigates LPS-induced neuroinflammation

**Fig. 2:**
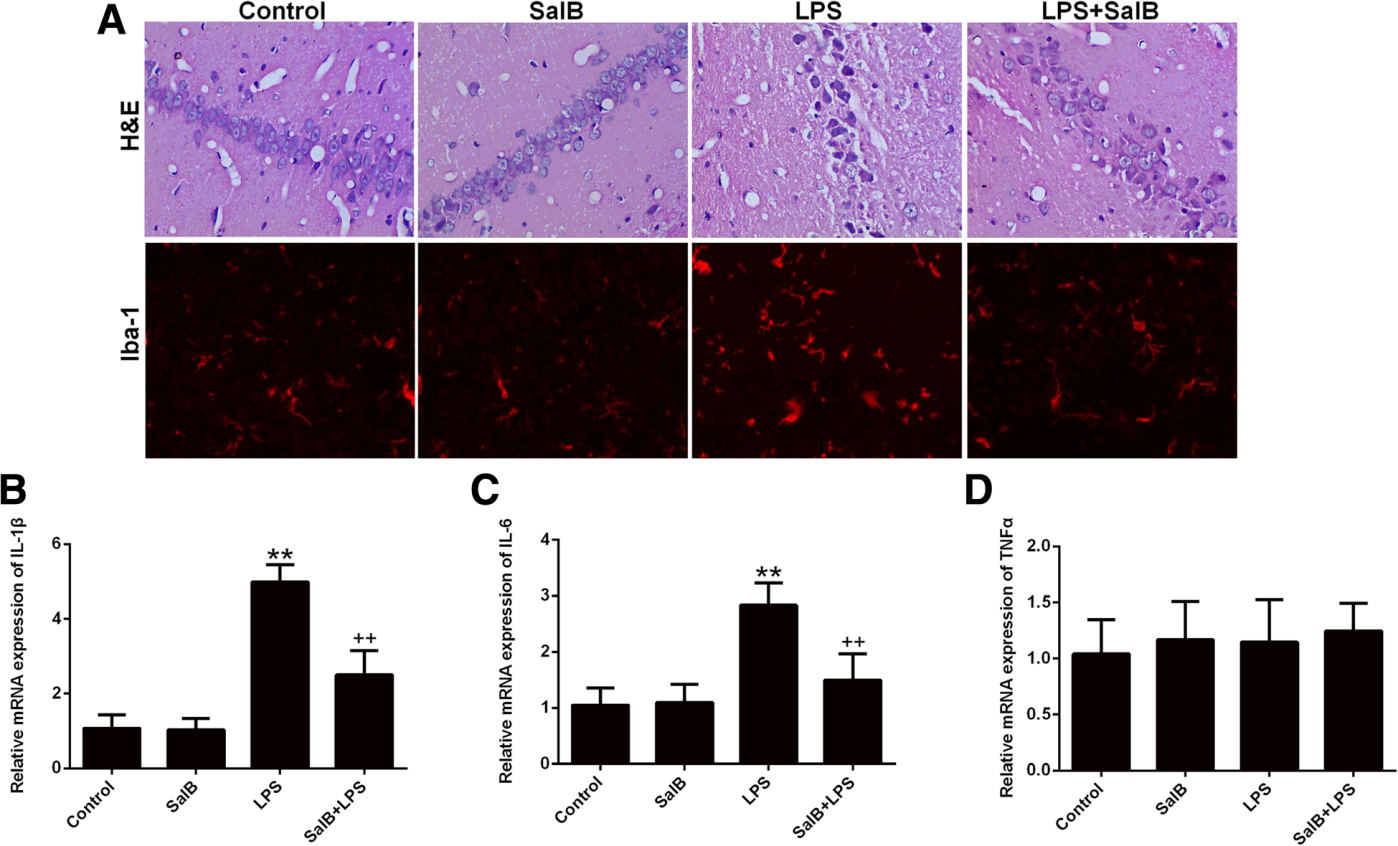
Neuroprotective effects of SalB against LPS-induced neuroinflammation. **a** Histological changes and microglial activation (Iba-1 immunofluorescence) following LPS and SalB treatment. mRNA expression of the proinflammatory cytokines, IL-1β (**b**), IL-6 (**c**), and TNF-α (**d**). Data are means ± SD (*n* = 8). ^**^ *p* < 0.01 compared to the control group.^++^ *p* < 0.01 compared to the LPS group

The neuronal histopathological changes in the hippocampus were observed by H&E staining. As shown in Fig. 2a, there were no significant neuronal abnormalities in the control and SalB group, while the neurons in the hippocampus of the LPS-treated group showed nuclear condensation and acidophilic degeneration, which were significantly alleviated by SalB co-administration. Likewise, ionized calcium-binding adapter molecular 1 (IBA-1) staining was used for the analysis of microglia activation and our data showed that SalB treatment mitigated LPS-induced microglia activation (Fig. 2a). In accordance, SalB also alleviated the enhanced expression of proinflammatory cytokines, IL-1β and IL-6 (Fig. 2b, c), in LPS group except that both SalB and LPS exerted no effect on TNF-α expression (Fig. 2d).

### Prolonged LPS exposure impaired autophagy without affecting autophagic flux

**Fig. 3:**
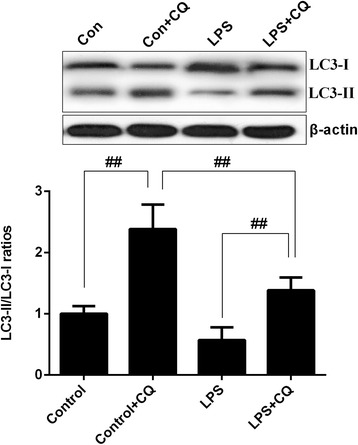
Representative western blot and quantitative analysis of LC3-II/I ratio in the absence or presence of chloroquine (CQ). Pretreatment with CQ induced an additional increase in the LC3-II/I ratio in both the control and LPS groups. Data are means ± SD (*n* = 6)

Given the critical role of autophagy in the regulation neuroinflammation, we further assessed the effects of LPS on the brain autophagic process. In accordance with previous findings [19], the western blot results showed that prolonged LPS exposure impaired autophagy with significant decrease of LC3-II/I ratio (Fig. 3, *p* < 0.01). To further examine whether the LPS-induced decrease of LC3-II/I ratio is due to impaired autophagosome formation or enhanced autophagic degradation, we blocked the autophagosome-lysome fusion by using the lysosomal inhibitor, CQ, following repeated LPS stimulation. As depicted in Fig. 3, CQ administration markedly increased the LC3-II/I ratio in both the control and LPS group, and LC3-II/I ratio was higher in the control + CQ group compared with the LPS + CQ group, indicating that sustained LPS treatment impaired autophagy by inhibiting autophagosome formation without affecting autophagic flux.

### SalB induced autophagy and attenuated LPS-induced NLRP3 inflammasome activation

**Fig. 4:**
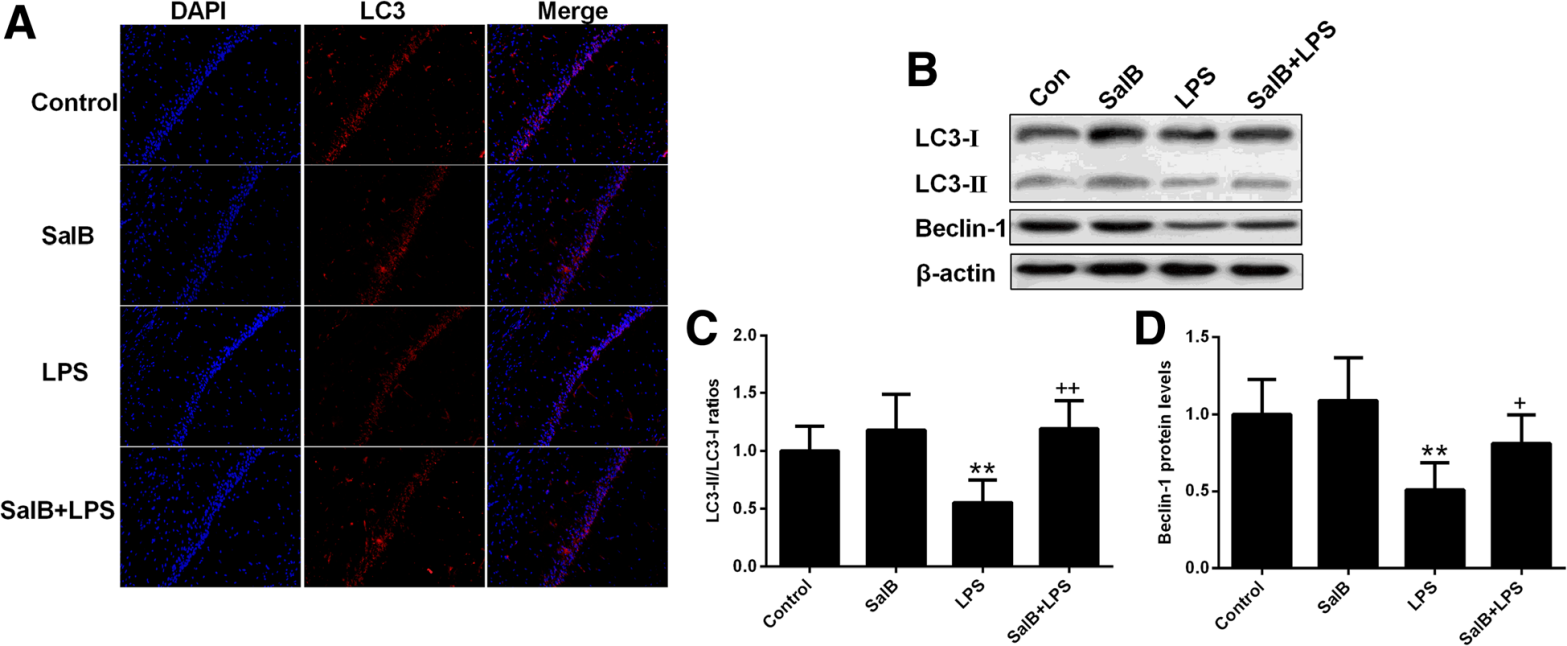
SalB restores LPS-induced impairment in autophagy. **a** Representative images of immunofluorescence assays of LC3 in the hippocampal CA1 region. Representative blots (**b**) and statistical graphs of relative LC3-II/I ratio (**c**) and Beclin-1 expression (**d**). Data are means ± SD (*n* = 8). ^**^ *p* < 0.01 compared to the control group. ^+^ *p* < 0.05, ^++^ *p* < 0.01 compared to the LPS group

SalB was recently proven to contain neuroprotective and autophagic modulating properties. As shown in Fig. 4a, the immunofluorescence analysis showed that LPS decreased LC3 expression, further confirming the inhibitory effect of sustained inflammatory state on autophagosome formation, which was restored by SalB treatment. In addition, our data also showed that although LPS decreased the expression of LC3-II/I ratio (*p* < 0.01) and Beclin-1 (*p* < 0.01), SalB facilitated autophagy in the brain of LPS-treated rats (Fig. 4b–d).

**Fig. 5:**
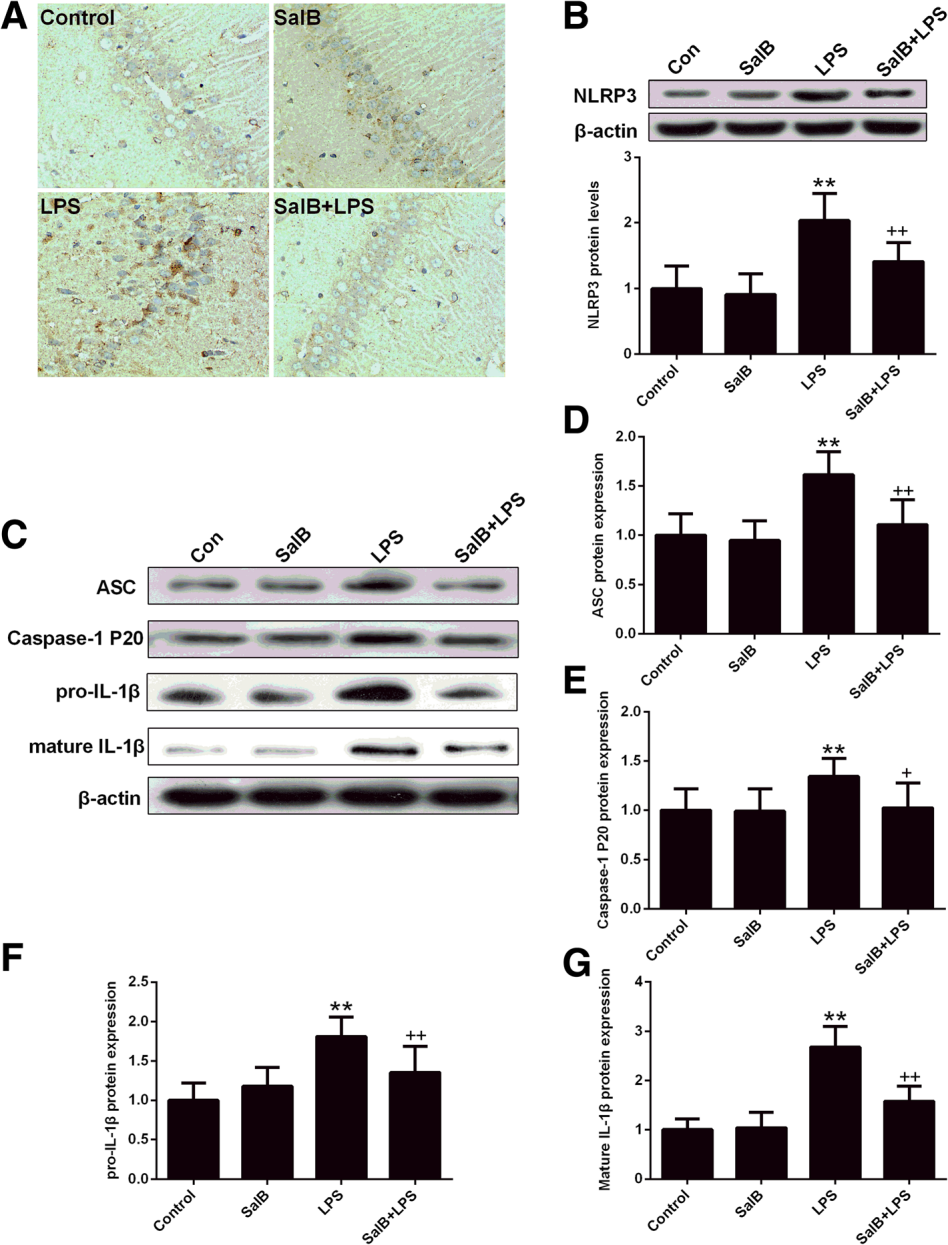
SalB ameliorates LPS-induced NLRP3 overexpression. **a** Representative images of immunohistochemical assays of NLRP3 in the hippocampal CA1 region. **b** Representative blots and statistical graphs of NLRP3. **c** Representative blots of NLRP3 inflammasome components (ASC and caspase-1 P20) and IL-1β. Statistical graphs of relative protein expression of ASC (**d**), caspase-1 P20 (**e**), pro-IL-1β (**f**), and mature IL-1β (**g**). Data are means ± SD (*n* = 8). ^**^ *p* < 0.01 compared to the control group. ^+^ *p* < 0.05, ^++^ *p* < 0.01 compared to the LPS group

Next, we analyzed the expression of NLRP3 inflammasome components (Fig. 5). We found that the protein levels of NLRP3 (Fig. 5a, b, *p* < 0.01) and ASC (Fig. 5d, *p* < 0.01) were significantly increased in the hippocampus of the LPS-exposed rats compared with that of the control group. In parallel, LPS treatment also induced significant activation of caspase-1 (cleaved caspase-1 P20) (Fig. 5e, *p* < 0.01) and enhanced the protein expression of both pro-IL-1β (Fig. 5f, *p* < 0.01) and mature IL-1β (Fig. 5g, *p* < 0.01), whereas SalB partly normalized the NLRP3 inflammasome activation with significant decrease of the protein levels NLRP3 inflammasome components (NLRP3, ASC, caspase-1 P20) and attenuated IL-1β activation compared with the LPS-treated group. These results suggest that autophagy process may interact with NLRP3 inflammasome to promote depression-like behaviors and neuroimmune activation, which may also actively participate in the molecular mechanisms of antidepressant effects of SalB.

## Discussion

Although accumulating evidence suggests the essential role of autophagy in depression, the underlying mechanism remains unknown. Given that autophagy serves as a protective mechanism in unfavorable conditions, such as starvation and inflammation, it is very likely that the interaction between autophagy and neuroimmune system is involved in both onset and treatment of depression. Autophagy limits detrimental and uncontrolled inflammation, through the clearance of inflammasomes and proinflammatory cytokines, thus functioning as a central fulcrum that balances inflammatory responses [20]. Disruption of autophagy process either by chemical compounds or genetic modulation enhances NLRP3 activation and the secretion of pro-inflammatory cytokines upon LPS stimulation [21, 22]. At the opposite, induction of autophagy by rapamycin leads to the degradation of NLRP3 and reduced amount of IL-1β [23].

To firstly provide the direct evidence linking the interaction between autophagy and neuroimmune system to depression, we assessed the expression of autophagic biomarkers, including LC3-II/I and Beclin-1, in the hippocampus of rats following sustained immune stimuli. As previously reported [15], prolonged exposure to LPS successfully induced anxiety and depression-like behaviors in rats, which resembles the clinical features that patients with depression are frequently under chronic subclinical inflammatory state. The continuous neuroinflammation also suppressed the expression of autophagic markers, LC3-II/I and Beclin-1. Notably, our data showed that chronically LPS-treated rats displayed unchanged hippocampal TNF-α mRNA expression, which is contradictory to the previous findings that the LPS-induced activation of toll-like-receptor 4 (TLR4) and NF-κB signaling transcriptionally and translationally promotes TNF-α expression [24]. However, in line with our findings, a recent study found that although the expressions of pro-inflammatory cytokines are preferentially enhanced in monocytes adapted by lower doses of LPS, the higher doses of LPS suppresses or has no effect on the inflammatory mediators, indicating that excessive or sustained TLR4 activation may result in endotoxin tolerance [25]. Similarly, this phenomenon was also recently documented in the central nervous system by showing that in spite of the significant increase of the pro-inflammatory cytokines in rat hippocampus following acute LPS administration, 3 months of LPS treatment only increases IL-1β protein levels but has no effect on IL-6 and decreases TNF-α status, implying that IL-1β could be the critical cytokine that accounts for the neruoinflammatory response and depression-like behaviors induced by repeated LPS stimulation [19].

Inflammasomes are intracellular signaling platforms, detecting a series of substances emerging during infections, cellular damage, or metabolic disturbances and thereby proteolytically activating the highly proinflammatory cytokines, IL-1β, and IL-18, whereas autophagy acts as a negative regulator of inflammasomes [9]. The activation of inflammasomes, especially NLRP3, contributes to the depression-like behaviors in animals caused by either stress or LPS. Genetic knock-out of NLRP3 increases the resilience to stress and blocks LPS-induced inflammatory responses [10, 26]. The autophagy protein, P62, can interact with the inflammasome component ASC, redirecting the ASC-containing inflammasome towards autophagosome and eventually delivered to lysosomes for destruction [27]. Additionally, it has been shown that IL-1β is sequestered in the LC3-positive autophagosomes upon TLR stimulation, and pro-IL-1β protein levels decreased when autophagy is induced by rapamycin, suggesting that pro-IL-1β is also targeted to autophagosome for degradation [22]. Since the NLRP3 inflammasome is one of the convergent pathways common to depression and autophagy, we further investigated the involvement of NLRP3 in the progression and alleviation of depression. Our data showed that sustained LPS treatment profoundly induced NLRP3 inflammasome activation with increased expression of the key components (NLRP3, ASC, caspase-1 P20), and IL-1β activation. TLR activation has been reported to promote both pro-IL-1β expression and degradation by autophagy, thus limiting the inflammation reaction due to minor insult [22]. However, we found that prolonged LPS exposure seems to induce exhaustion of autophagy, resulting in aberrant pro-IL-1β production and NLRP3 inflammasome activation. These data are consistent with previous findings that 0.5 mg/kg LPS treatment for 3 months impairs autophagy in rat brain [19]. Similarly, Kim and colleagues also found that LPS stimulation suppresses LC3II accumulation and enhances inflammasomes activation in murine peritoneal macrophages [28]. These contradictory results may be due to the experimental conditions, such as cell types and LPS doses and treatment duration. Considering that the LPS-induced decrease of autophagic markers could be attributed to either the impaired autophagic formation or enhanced autophagosome clearance, we further assessed the effect of LPS stimulation on autophagic flux in the animal brain. The pronouncedly increased LC3-II/I ratio in both control and LPS group following CQ administration suggests that autophagic flux was normal in LPS-treated rats. Likewise, the evidence concerning the impact of LPS on autophagic flux is limited and controversial. Although most of the studies are in support of our findings showing that LPS exposure enhances or has no effect on autophagic flux [28, 29], Liu et al. found that LPS treatment suppresses autophagic flux and activates NLRP3 inflammasome in Kupffer cells [30]. Therefore, it would be interesting for future studies to evaluate the autophagic process in the brain following acute or prolonged challenge of LPS given the essential role of the interaction between autophagy and inflammation in neurological functions.

Meanwhile, we also found that SalB facilitated autophagy and attenuated LPS-induced NLRP3 activation. Although the mechanisms are yet not fully understood, autophagy can directly sequester and degrade inflammasome components or indirectly clear endogenous danger signals that induce inflammasome formation, such as reactive oxygen species and DNA. Additionally, it should be noted that SalB can activate nuclear factor erythroid 2-related factor 2 (Nrf2) signaling and promote the clearance of ROS [29], which might be also involved in the restoration of NLRP3 overactivation. In line with our findings, a recent research also demonstrated that Beclin-1 is suppressed and mitochondrial autophagy is compromised in learned helplessness depression model, which is alleviated by fluoxetine treatment [31]. Similarly, a number of antidepressant drugs were recently shown to enhance autophagy in vitro and in vivo [32]. Lithium, the prototypic mood stabilizer, and other antidepressant strategies, such as electroconvulsive seizures (ECS), also can promote brain autophagy process [33, 34]. Additionally, the mTOR inhibitor and classical autophagy inducer, rapamycin, confers antidepressant effects through promoting autophagy [7, 35]. Although it has been generally accepted that the induction of autophagy promotes antidepressant actions and some of the antidepressants can induce autophagy beyond their influence on monoaminergic neurotransmission, it should be noted that not all antidepressants may act the same way on autophagy. It was found in a recent study that while amitriptyline and citalopram facilitate autophagic processes in rat primary astrocytes and neurons, venlafaxine fails to affect autophagy [36]. Intriguingly, desmethylclomipramine blocks autophagic flux in tumor cells, thus potentiating the cytotoxic effect of chemotherapeutic agents [37, 38]. Based on these evidence, it seems likely that autophagy might be involved in the pharmacological actions of some antidepressants and the impact of antidepressants on autophagic process may differ according to the selected drugs and cell types.

SalB has been proven to provide anti-inflammatory, antidepressant, and neuroprotective activities. Recently, several lines of evidence suggest that SalB is a novel autophagy inducer, whereby to exert cardio protective and antitumor activities [13, 39, 40]. To explore whether the facilitating effect of SalB on autophagy process is involved in its antidepressant and neuroimmune modulating effects, we assessed the effects of SalB on autophagic biomarkers in the inflamed brain. Our data firstly showed that SalB mitigated the LPS-induced behavioral changes, microglial activation, and inflammatory responses and restored the compromised autophagy process. In support to our data, a recent study showed that SalB effectively ameliorates chronic stress-induced depressive-like behaviors and neuroimmune activation [14]. The interrelationship between autophagy and neuroimmune actions is intricate, and SalB has complex pharmacological effects, such as anti-inflammatory and antioxidant activities [29]. It should be noted that although the modulating effect of SalB on autophagy may contribute to the normalization of NLRP3 inflammasome in SalB treatment group, other mechanisms, such as ROS scavenging and direct neuro-immunomodulating activities of SalB, might also be involved in the restoration of NLRP3 overactivation and neuroinflammation. Thus, the potential central role of autophagy in the antidepressant and anti-inflammatory actions of SalB warrants further direct examination.

## Conclusions

The evidence concerning the interrelationship among neuroinflammation, autophagy, and depression is interesting and blank. Collectively, our data firstly provide the evidence showing that the LPS-induced sustained inflammatory state may interact with autophagy process to promote behavioral deficits, whereas SalB enhanced autophagy and induced the clearance of excessive NLRP3, resulting in antidepressant and neuroprotective effects. Although the underlying mechanisms need to be further investigated, our research highlighted the synergism between autophagy and neuroimmune system in the progression and remission of depression and raise the possibility that SalB could be a promising naturally occurring compound in the treatment of neuropsychiatric disorders.

## Abbreviations

ASC: Apoptosis-associated speck-like protein containing a caspase recruitment domain
CQ: Chloroquine
FST: Forced swim test
IBA-1: Ionized calcium binding adapter molecular 1
LPS: Lipopolysaccharide
NF-κB: Nuclear factor kappa B
NLRP3: Nod-like receptor pyrin containing 3 inflammasome
SalB: Salvianolic acid B
SPT: Sucrose preference test
SSRI: Selective serotonin reuptake inhibitors
